# Characteristics of bacterial communities in rhizosphere and bulk soil in Fe-deficient citrus growing in coastal saline-alkali land

**DOI:** 10.3389/fpls.2023.1335843

**Published:** 2024-02-20

**Authors:** Tianchi Jiang, Jiuzhou Chen, Yu Huang, Xiaoyan Chang, Yuping Wu, Gaoping Liu, Runze Wang, Kuan Xu, Lingli Lu, Haizhong Lin, Shengke Tian

**Affiliations:** ^1^Ministry of Education (MOE) Key Laboratory of Environmental Remediation and Ecological Health, College of Environmental and Resource Sciences, Zhejiang University, Hangzhou, China; ^2^Xiangshan Agricultural and Rural Bureau, Ningbo, China; ^3^Ningbo Agricultural and Rural Bureau, Ningbo, China; ^4^Huangyan Agricultural and Rural Bureau, Taizhou, China; ^5^Agricultural Technology Extension Center of Huangyan District, Taizhou, China

**Keywords:** citrus, rhizosphere, bacterial community, saline-alkali land, nutrition

## Abstract

**Aims:**

Citruses often occur with imbalance in iron nutrition in coastal saline-alkali lands, which severely limits the yield and quality of the fruit. In the rhizosphere, the salt content plays a crucial role in reducing uptake of iron, as well as the activity and abundance of bacteria. However, few studies have explored how salt content affects the effectiveness of iron and the community structure of bacteria across different vertical spatial scales.

**Methods:**

We investigated the citrus rhizosphere (0–30 cm) and bulk (0–60 cm) soil microenvironments of the coastal saline soil were analyzed using the 16S rRNA amplicon and inductively coupled plasma-optical emission spectroscopy.

**Results:**

We found that the nutrient-related elements in the rhizosphere and bulk soil decreased with increasing soil depth, while the salinity-related elements showed the opposite trend. The nutrient-related element content in the rhizosphere was higher than that in the bulk, whereas the salinity-alkaline-related element content was lower than that in the bulk. The structure and diversity of bacterial communities are affected by the rhizosphere and soil depth. In the bulk, there are enriched bacteria such as WB1-A12, Nitrospiraceae and Anaerolineae that are tolerant to salt-alkali stress. In the rhizosphere, bacteria that promote plant nutrient absorption and secretion of iron carriers, such as *Pseudomonas*, *Streptomyces*, and *Duganella*, are prominent.

**Conclusions:**

The soil depth and rhizosphere affect soil nutrients and saline alkali-related factors. Changes in soil depth and rhizosphere determine the structure and diversity of bacterial communities. Rhizosphere enhances iron absorption promoting bacteria to alleviate iron deficiency stress in saline-alkali soils. Our results indicate that citrus roots maybe can resist the stress of iron deficiency in saline-alkali soils by enhancing iron absorption promoting bacteria.

## Graphical Abstract

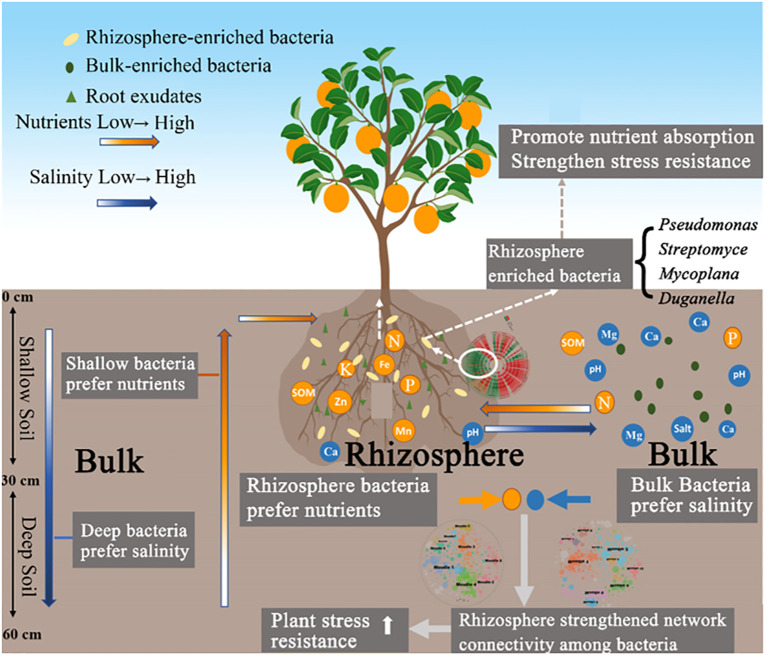

## 1 Introduction

Citrus fruits are one of the largest fruit categories in terms of global production. However, iron nutrition imbalance in citrus often occurs in coastal saline-alkali soils, and the lack of scientific nutrient management measures have severely restricted the improvement of the fruit yield and quality (Storey and Walker, 1998). In coastal saline soils, although the total iron content in the soil is high, the availability of iron may be an issue. Owing to the high salt content and pH of such soil, iron mostly exists in the form of insoluble iron hydroxide (Guerinot and Yi, 1994). To cope with iron deficiency stress and enhance iron absorption, plants use two mechanisms, namely, mechanisms I and II (Römheld, 2000). In Strategy I plants, at first the Fe^3+^ complexes from soil are solubilized through H^+^ efflux-mediated rhizosphere acidification; second, the resulting Fe^3+^ is reduced to Fe^2+^, a process that depends on FERRIC REDUCTION OXIDASE 2 (FRO2); third, the Fe^2+^ is subsequently transported into root cells by the divalent metal transporter IRON-REGULATED TRANSPORTER 1. By contrast, Strategy II plants, represented by wheat monocots, directly take up Fe^3+^ by secreting Fe^3+^ chelating substances. Citrus is a plant that uses mechanism I. However, the effect of mechanism I alone is not sufficient to help citrus plants cope with iron deficiency.

Soil microorganisms are also important regulators of plant productivity (Chandra et al., 2021 and Thakur et al., 2023). In a nutrient-deficit environment, microbial–plant symbiosis greatly contributes to

obtaining sufficient nutrients (Van der Heijden et al., 2008). For example, siderophores produced by rhizosphere microorganisms have a stronger Fe^3+^ affinity than plant siderophores and can chelate Fe^3+^ that can be directly absorbed by plants (Marschner et al., 2011). Fe resource acquisition are potential functions of the core rhizomicrobiome of the wild rice Oryza rufipogon (Chang et al., 2022). Studies have shown that plants grown in sterilized soil show obvious symptoms of iron deficiency and chlorosis, whereas those in unsterilized soil grow well with high iron content in the roots, which proves that the existence of microorganisms is necessary for plant iron nutrition (Masalha et al., 2000).

In addition to producing siderophores, rhizosphere microorganisms can acidify the soil, secrete phenolic-reducing substances, or use Fe^3+^ as an electron acceptor to reduce it to Fe^2+^, thereby increasing the availability of iron in the soil (Pii et al., 2015). Therefore, rhizosphere microorganisms play a vital role in obtaining iron from alkaline coastal saline soils with high salt content.

Current research on soil microbial communities focus on cultivated soil (0–20 cm), while investigations on the characteristics of deep soil microbial communities are still lacking (Xu et al., 2021). Although existing studies have shown that the biomass and diversity of microorganisms decrease significantly with an increase in soil depth (Stone et al., 2014), the soil below 20 cm still contains approximately 35% of the total microbial biomass, which plays an important role in the ecological functions of the soil (Jiao et al., 2018). Moreover, there are new and undescribed microorganisms in the deeper soils with unique life history strategies and functions, such as salt tolerance and alkali tolerance (Brewer et al., 2019).

Therefore, this study is based on the soil of a citrus orchard in coastal saline-alkali land with high salt content, where citrus is prone to iron deficiency. We combined inductively coupled plasma-optical emission spectroscopy (ICP-OES) with 16S rRNA amplicon technology to analyze the environmental factors and microbial structures of the rhizosphere and bulk soil of a citrus orchard in coastal saline-alkali land. We wanted to explore (i) the distribution and structural characteristics of soil bacterial communities in coastal saline-alkali soils at different soil depth; (ii) the difference in the bacterial community structure between citrus rhizosphere and bulk soil; and (iii) the variation in the responses of citrus rhizosphere and bulk soil bacterial communities and microbial networks to environmental factors.

## 2 Materials and methods

### 2.1 Sampling location and soil collection

We collected soil from a citrus orchard having coastal saline-alkali land located in the town of ShiPu in Ningbo City, Zhejiang Province, China (29°15′57″N, 121°55′56″E). The soil was alkaline with a pH of 7.64, electrical conductivity was 1.70 ds·m^−1^, and salt content was high. More details of the soil physicochemical properties are provided in the **Supplementary Material** (**Supplementary Table S1**).

We selected six Beni-Madonna citrus trees with similar growth and excavated their soil profile of 0–60 cm depth under the drip line of the canopy. The six trees were collected from citrus greenhouse in Shipu Town in Ningbo City, Zhejiang Province, China (29°15′57″N, 121°55′56″E). They are situated within the same orchard. Taking 10 cm as a soil layer, we collected 0–60 cm bulk soil and 0–30 cm rhizosphere soil (no roots below 30 cm), immediately placed them in an ice box, and brought them back to the laboratory for determination of the physical and chemical properties. When collecting the rhizosphere soil for extracting soil microbial RNA, by extending the growth direction of the root system, we dug out fine roots (2–3 mm in diameter), gently shook off the loose soil on the roots, and placed the root system together with the soil attached to it. It was placed into a centrifuge tube containing 50 mL of pre-cooled sterile phosphate-buffered saline (PBS) and agitated several times. The above operations were performed under 4°C conditions, and the sampling time for each sample did not exceed 5 min to prevent soil microbial DNA degradation. After shaking and washing, the root system was removed with sterile tweezers and the soil was preserved. Bulk soil was collected from soil far from the trees and without any root distribution. The samples were placed in an incubator containing dry ice and brought back to the laboratory for follow-up operations under low-temperature conditions (Edwards et al., 2015).

### 2.2 Determination of soil physical and chemical properties

The air-dried soil sample was passed through a 20-mesh sieve and extracted with CO_2_^−^ removed ultrapure water at a soil-water ratio of 1:5 to determine water-soluble base ions in the soil. The cations were measured by ICP-OES (Thermos 6300, USA), while Cl^−^, SO_4_^2−^, NO_3_^−^, and other anions were measured by ion chromatography (Thermo Scientific, USA). Soil electrical conductivity (EC) was measured using a conductivity meter in the soil sample extracted with ultrapure water at a water-soil ratio of 5:1 after passing through a 20-mesh sieve.

To analyze the concentrations of total Fe and other nutrient elements, soil samples (0.1 g) were digested with 5 mL HNO_3_, 1 mL HClO_4_, and 1 mL HF at 180°C for 10 h. Bio-available Fe and other nutrient elements were extracted with diethylene triamine penta acetic acid (DTPA) extracting agent (0.005 mol/l DTPA, 0.01 mol/L CaCl_2_, and 0.1 mol/L triethanolamine; pH 7.3). Soil samples were analyzed for DTPA-extractable metals in a 1:2 soil:solution mixture, which was obtained after shaking for 2 h. Concentrations of elements in the digestive and extractive solutions were determined using ICP-OES (Agilent 7500a, USA). Sample replicates, reagent blanks, and soil standard reference material (GBW07429, National Research Center for Certified Reference Materials of China) were included in each batch of analysis to ensure quality of the analysis.

The alkali-hydrolyzed nitrogen was determined using the alkali-hydrolyzed diffusion method. The methods of extracting available phosphorus (OP) from acidic, neutral, and alkaline soils were different. For acid soil (pH < 6.5), a mixed extraction solution of 0.05 mol/L HCl and 0.025 mol/L H_2_SO_4_ was used; whereas for neutral and alkaline soils (pH > 6.5), 0.5 mol/L NaHCO_3_ was used and phosphorus was determined by the molybdenum-antimony anti-colorimetric method. The available potassium was extracted with 1 mol/L NH_4_OAc, and the extracted solution was analyzed using a flame spectrophotometer. Exchangeable calcium and magnesium ions were extracted with 1 mol/L NH_4_OAc and analyzed using ICP-OES.

### 2.3 Soil DNA extraction, 16S rRNA gene amplification and MiSeq sequencing

The soil samples were centrifuged at 4°C at 1,000 rpm for 5 min, the supernatant was discarded, and the remaining soil sample was used for soil microbial RNA extraction and sequencing. DNA was extracted from 0.5 g of soil from each sample according to the instructions provided in the Power Soil DNA Isolation Kit (MOBIO, USA). NanoDrop TM ND 2000 (PEQLAB biotechnology GmbH, Ebersberg, Germany) was used for DNA quality testing. A 20 μL Polymerase Chain Reaction (PCR) amplification system was used, and the reaction conditions were: pre-denaturation at 95°C for 3 min, denaturation at 95°C for 30 s, annealing at 55°C for 30 s, extension at 72°C for 45 s for 27 cycles, final extension for 10 min, and the sample was purified. The MiSeq (Illumina, CA, USA) sequencing platform was used for the PCR amplified products, the primers used for sequencing and amplifying the 16SrRNA gene in the V3-V4 region were:

341F:5′- CCT AYGGGRBGCASCAG-3′

805R:5′- GGACTACNNGGGTATCTAAT-3′

### 2.4 Sequencing data processing and analysis

A total of 1,481,011 high-quality sequences from 54 samples (27,426 sequences each on average) were generated using high-throughput sequencing, and the number of reads in each sample ranged from 3,282 to 47,250. To perform quality control analysis of the high-quality sequences and eliminate redundancy QIIME2 was used. After removing the chimera, 10,503 characteristic species sequences were obtained. The sequences were clustered into amplicon sequence variants (ASVs) with 97% similarity using the DADA2 method and the species were annotated using the SILVA database.

QIIME2 was used to calculate the classification and phylogenetic diversity indices within the samples. The Bray–Curtis distance was used to estimate the diversity index based on taxonomy and systematic genetics. The “PcoA” function of the “ape” package in R (version 3.6.0) was used to perform principal coordinate analysis (PcoA) on the overall structure of the bacterial communities. Cluster analysis was performed based on the Bray–Curtis differences among the samples. The factorial Kruskal–Wallis rank sum test (α = 0.05) was used for linear discriminant analysis (LDA) to determine the species that were significantly different between the treatments. Statistically significant species were used to generate taxonomic branch maps to illustrate the specific species differences between the rhizosphere and bulk soil. Redundant analysis (RDA) was performed using the Vegan package to determine the impact of environmental factors on the structure of microbial communities. “Pheatmap” package was used to draw a heat map of the correlation between specific strains and environmental factors.

Using MENA, a molecular ecological network (MENs) of the citrus rhizosphere and bulk soil was constructed based on the random matrix theory (RMT) (http://ieg4.rccc.ou.edu/mena). The correlation between the constructed bacterial groups was restricted according to the adjusted p values of the maximal information coefficient (MIC) and false discovery rate (FDR). Through the method based on random matrix theory (RMT), the cut-off thresholds of the rhizosphere and bulk soil were determined to be 0.83 and 0.82 respectively, and the visualization is presented by Gephi. A greedy modular optimization method was adopted for module separation. The Vegan package was used to perform manual inspection, analyze the species information of each module and the corresponding environmental factors, and the chart was drawn using the ggcor package.

### 2.5 Statistical analysis of data

The data in the chart represent the average ± standard deviation of 6–8 individual plants. The data were analyzed for significant difference using SPSS20. Two samples were compared using an independent sample T test, and analysis of variance (ANOVA) was used to analyze the significance of more than three samples. Spearman’s correlation analysis was used for correlation analysis; the significance level was P < 0.05, and extremely significant level was P < 0.01. The chart was drawn using Origin 8.

## 3 Results

### 3.1 Differences in the physical and chemical properties of rhizosphere and bulk soil

The results showed that the nutrient content of organic matter (SOM), alkali-hydrolysable nitrogen (AN), available potassium (AK), and available zinc (Azn) in the rhizosphere and bulk soil decreased significantly with increasing soil depth. However, changes in the pH, sodium ions (Na^+^), exchangeable calcium (ExCa), and magnesium ion (Mg^2+^) showed opposite trends. In addition, EC, Cl^−^, and SO_4_^2−^ increased significantly only in the bulk soil with increasing depth and no significant difference was observed in the rhizosphere (**Supplementary Table S2**). It was observed in both the rhizosphere and bulk soil that the effective nutrients in the soil showed surface accumulation, while the elements related to alkalinity and salinity accumulated in the bulk and deep soil (below 30 cm). Salt-alkali stress may be one of the reasons why citrus roots cannot grow in soil below 30 cm.

The contents of nutrient-related elements, such as OP and AK, in the rhizosphere were significantly higher than those in the bulk, while the contents of saline-alkali-related elements, such as pH, Na^+^, and exchangeable magnesium (ExMg), were significantly lower than those in the rhizosphere (**Table 1**). It is believed that the existence of the root system weakens the rhizosphere microenvironment in terms of salinity and alkalinity, and thus, increases the content of available nutrients.

**Table 1:** Physicochemical properties of citrus rhizosphere and bulk soil in coastal soil profile.

Analysis index	Rhizosphere(depth cm)			Bulk(depth cm);					
	0-10	10-20	20-30	0-10	10-20	20-30	30-40	40-50	50-60
**SOM(g·kg^-1^);**	23.08 ± 1.20 a’	19.13 ± 1.86 b’	11.01 ± 1.05 c’	20.72 ± 1.64 a	20.33 ± 0.62 a	7.69 ± 0.74 b	7.47 ± 0.75 b	7.91 ± 0.83 b	7.28 ± 1.14 b
**AN(mg·kg^-1^);**	103.79 ± 13.24 a’	54.36 ± 7.67 b’	35.71 ± 9.87 b’	61.90 ± 9.55 a	49.81 ± 11.31 b	28.45 ± 3.01 c	18.90 ± 1.50 cd	16.27 ± 0.76 d	16.09 ± 0.86 d
**OP(mg·kg^-1^);**	35.60 ± 4.21 a’	18.15 ± 2.47 b’	30.91 ± 2.44 a’	20.37 ± 0.97 a	18.50 ± 2.50 a	12.05 ± 3.17 b	11.59 ± 2.89 b	11.05 ± 0.54 b	11.67 ± 2.96 b
**AK(mg·kg^-1^)**	554 ± 76.23 a’	400.67 ± 34.68 b’	334 ± 37.03 b’	383 ± 35.95 a	332 ± 36.12 b	305.5 ± 26.08 bc	288.8 ± 16.12 bc	280.8 ± 27.15 c	262.5 ± 10.78 c
**pH**	7.86 ± 0.29 b’	8.31 ± 0.30 a’	8.45 ± 0.10 a’	8.45 ± 0.13 b	8.41 ± 0.13 b	8.64 ± 0.20 ab	8.69 ± 0.21 ab	8.73 ± 0.25 a	8.81 ± 0.24 a
**EC(ds·m^-1^);**	0.83 ± 0.07 a’	0.80 ± 0.16 a’	0.89 ± 0.09 a’	0.84 ± 0.08 c	0.98 ± 0.14 b	0.90 ± 0.15 c	1.04 ± 0.20 bc	1.25 ± 0.25 ab	1.44 ± 0.10 a
**Salt(g·kg^-1^);**	0.43 ± 0.04 a’	0.41 ± 0.09 a’	0.47 ± 0.05 a’	0.44 ± 0.05 c	0.52 ± 0.08 b	0.47 ± 0.09 c	0.55 ± 0.11 bc	0.67 ± 0.14 ab	0.78 ± 0.06 a
**ExK(mg·kg^-1^);**	476.76 ± 27.54 a’	328.86 ± 44.22 b’	288.55 ± 21.35 b’	288.76 ± 32.37 a	231.05 ± 19.38 b	205.16 ± 16.41 bc	174.77 ± 9.58 c	181.86 ± 12.52 c	193.27 ± 18.30 c
**ExNa(mg·kg^-1^);**	820.67 ± 65.19 a’	572.56 ± 9.27 b’	508.31 ± 32.51 b’	514.51 ± 42.46 a	457.65 ± 53.33 ab	390.76 ± 45.82 b	382.26 ± 20.07 b	460.89 ± 45.50 ab	485.39 ± 20.97 a
**ExCa(mg·kg^-1^);**	4968.29 ± 663.84 b’	5938.65 ± 443.97 b’	7573.88 ± 625.78 a’	5111.73 ± 65.90 c	6180.40 ± 719.76 b	7581.48 ± 761.57 a	7658.07 ± 565.51 a	7695.81 ± 546.88 a	7588.04 ± 504.65 a
**ExMg(mg·kg^-1^);**	424.65 ± 12.91 b’	434.80 ± 39.87 b’	548.85 ± 41.03 a’	508.71 ± 21.82 c	648.62 ± 32.20 b	698.51 ± 32.78 a	710.82 ± 24.50 a	695.06 ± 23.09 a	628.39 ± 25.04 b
**AvZn(mg·kg^-1^);**	3.06 ± 0.32 a’	0.96 ± 0.12 b’	0.79 ± 0.08 b’	2.92 ± 0.27 a	1.36 ± 0.13 b	0.74 ± 0.23 c	0.75 ± 0.15 c	0.74 ± 0.03 c	0.79 ± 0.06 c
**AvFe(mg·kg^-1^);**	14.04 ± 1.61 a’	14.89 ± 2.84 a’	13.70 ± 4.14 a’	13.30 ± 0.96 ab	16.74 ± 1.31 a	10.03 ± 2.95 bc	9.75 ± 0.97 bc	8.06 ± 1.077 c	11.32 ± 1.62 bc
**AvMn(mg·kg^-1^);**	23.04 ± 1.24 a’	22.55 ± 2.97 a’	22.39 ± 3.17 a’	32.71 ± 2.80 a	31.99 ± 3.05 a	23.18 ± 3.24 b	18.68 ± 1.91 c	18.18 ± 2.20 c	17.28 ± 1.60 c

The significant difference of soil depths is indicated with small letters (P < 0.05); SOM, soil organic matter; AN, available nitrogen; OP, available phosphorus; AK, available potassium; EC, electrical conductivity; ExK, exchangeable potassium; ExNa, exchangeable sodium; ExCa, exchangeable calcium; ExMg, exchangeable magnesium; AvZn, available Zinc; AvFe, available Iron; AvMn, available Manganese.

### 3.2 Differences in the structure of rhizosphere and bulk soil bacterial communities and its response to environmental factors in soil profile

#### 3.2.1 α diversity of citrus rhizosphere and bulk soil bacterial communities

**Figure 1:**
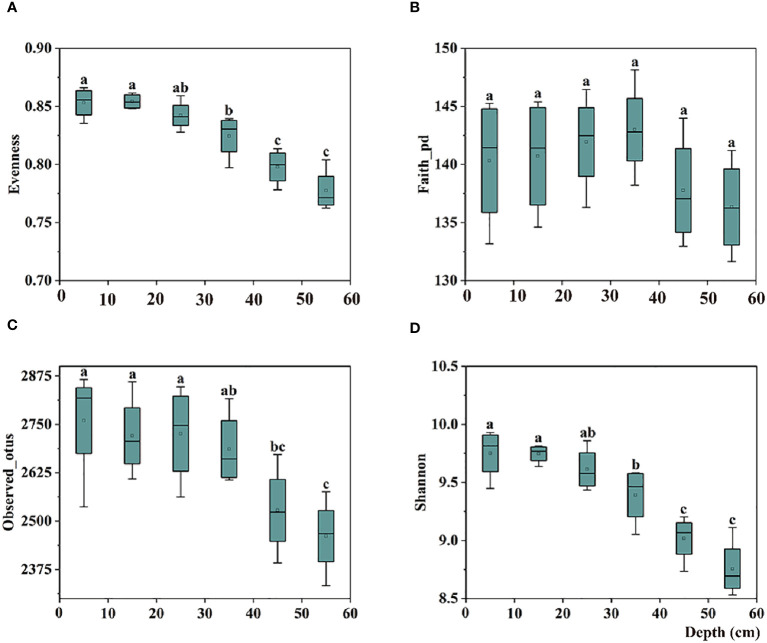
Box plots of bulk soil bacterial communities for alpha-diversity indices, including the **(A)** Evenness, **(B)** Faith-pd, **(C)** Observed-otus, and **(D)** Shannon indices in 0–60 cm soil. The significant difference in depth is indicated by small letters above the box at P < 0.05.

In the 0–30 cm soil profile, the alpha diversity of the bulk soil bacterial communities increased with depth; however, no significant difference was observed. In the 40–60 cm soil profile, the evenness index (evenness) and two richness indices (Observed_otus and Shannon) of the communities’ alpha diversity decreased significantly with increasing depth (**Figure 1**).

**Figure 2:**
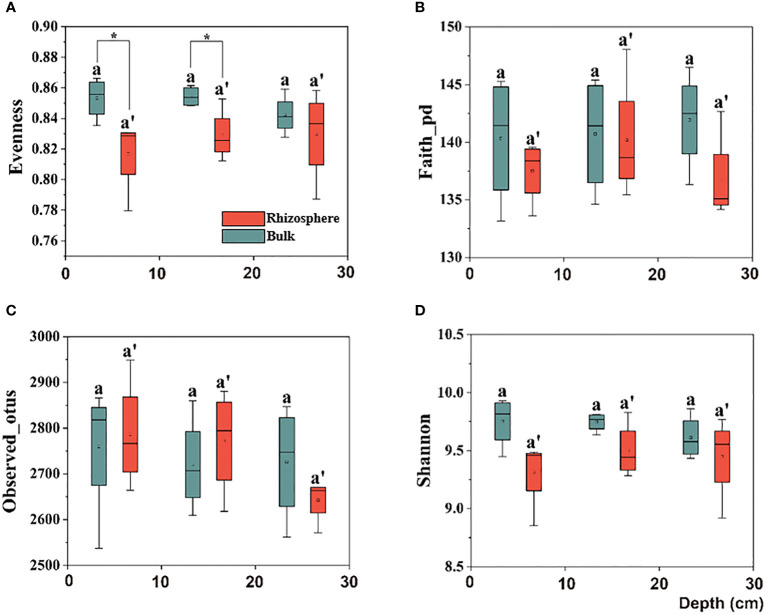
Box plots of citrus rhizosphere and bulk soil bacterial communities for alpha-diversity indices, including the **(A)** Evenness, **(B)** Faith-pd, **(C)** Observed-otus, and **(D)** Shannon indices in 0–30 cm soil. The significant difference in depth was indicated by small letters above box P < 0.05. The significant difference between the citrus rhizosphere and bulk soil at the same depth is indicated by an asterisk (*P < 0.05).

Further, upon comparing the difference in the alpha diversity of the citrus rhizosphere with the bulk soil bacteria in the 0–30 cm soil, similar to the bulk performance, no significant difference was observed. However, in the 0–20 cm soil layer, although there was no significant difference in the richness index between the rhizosphere and bulk soil, the evenness index of the rhizosphere bacteria was significantly lower than that of the bulk, which indicates that the rhizosphere soil environment makes the bacterial distribution more uniform (**Figure 2**).

#### 3.2.2 Bacterial community structure in the rhizosphere and bulk soil

**Figure 3:**
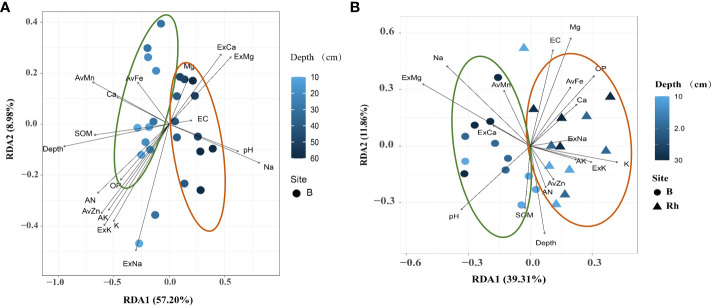
Principal coordinate analysis (PCoA) of the structure of the bacterial communities in **(A)** 0–60 cm bulk and **(B)** 0–30 cm rhizosphere soils based on the Bray–Curtis distances.

PCoA analysis showed differences in the bacterial community structure. In the 0–60 cm soil profile, there were differences in the structure of bulk soil bacterial communities at different depths, and the total explanations for the differences in the bulk community structure by depth was 38.89% (**Figure 3A**). In the 0–30 cm soil layer, depth was not the cause of the difference in community structure, but the micro-environment of the rhizosphere and bulk soil were the factor that affected the structure of the bacterial communities. The total explanation for the differences in the communities was 27.21% (**Figure 3B**).

### 3.3 Correlation between rhizosphere and non-rhizosphere bacterial communities and soil environmental variables

**Figure 4:**
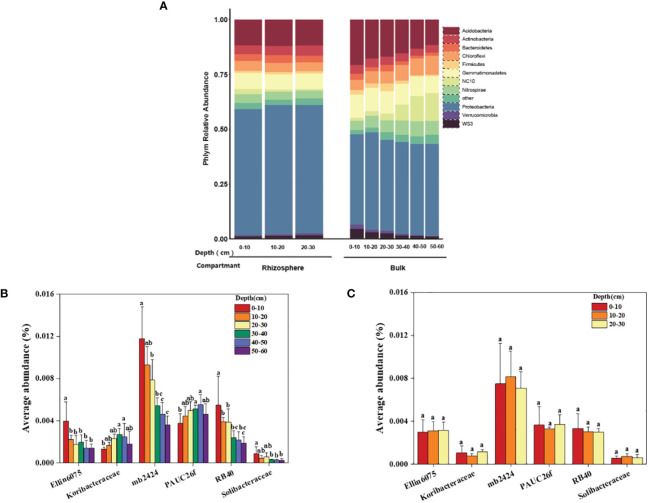
Redundancy analysis (RDA) ordination plots for the two principal dimensions of the relationship between the environmental parameters and bacterial communities in rhizosphere and bulk soil. The operational taxonomic units (OTUs) of each environmental variable are represented by a dot; the gradient of environmental parameters is shown in the note on the right; the percentage of variation is explained by each axis is shown. The relationship between the relevant environmental variables and RDA axis is represented by the length and angle of the arrow. **(A)** bulk soil of 0–50 cm; **(B)** rhizosphere and bulk soil of 0–30 cm.

The results showed that the bulk soil bacterial communities were affected by depth in response to soil environmental variables. The 0–30 cm bulk soil profile was mainly affected by nutrient-related variables, including SOM, AN, OP, AK, and available zinc (AvZn), iron (Fe), and manganese (Mn). While the 40–60 cm deep bulk soil was greatly affected by salinity- and alkalinity-related variables, including pH, Na^+^, ExCa, and ExMg (**Figure 4A**). In addition, the bacterial communities between the 0–30 cm rhizosphere and bulk soil were also affected by environmental variables. The bacterial communities in the rhizosphere soil were greatly affected by nutrient-related elements, including AN, OP, AK, AvZn, and available iron (AvFe). Bulk soil bacteria were mainly affected by salt-related elements, including pH, Na^+^, ExCa, and ExMg (**Figure 4B**).

Based on the above results, the citrus rhizosphere bacterial communities were mainly affected by nutrient variables, whereas those in the bulk soil were mainly affected by salinity-alkaline-related variables. The influence of salinity and alkalinity was directly proportional to the depth of the soil profile. The decreasing trend of relationship between bacterial communities and soil nutrients was as follows: rhizosphere area > bulk (0–30 cm) > bulk (30–60 cm), whereas the relationship between salinity and alkaline variables showed the opposite trend.

### 3.4 Differences in composition of bacterial communities between rhizosphere and bulk soil at different depths

**Figure 5:**
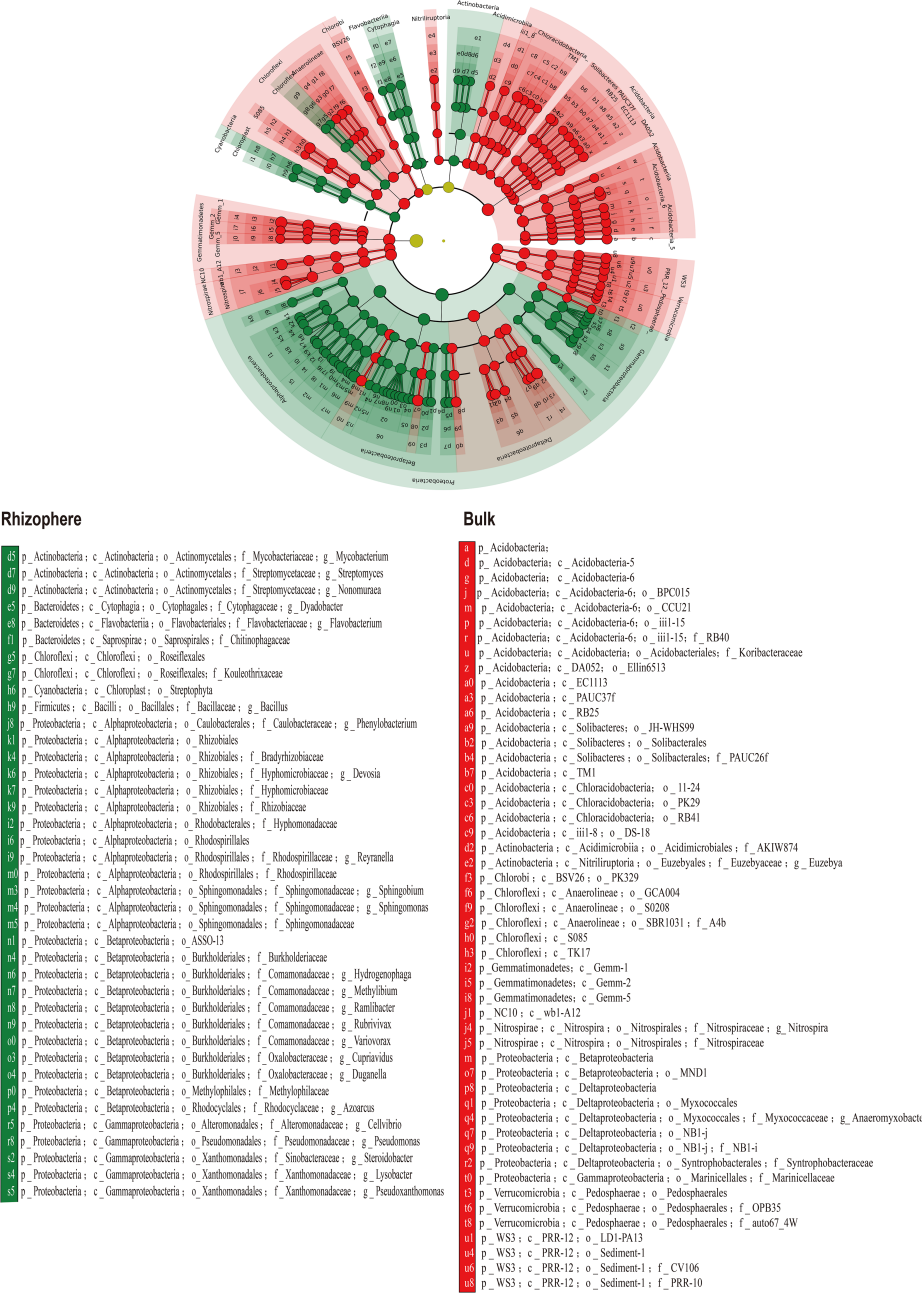
Dominant bacterial communities in the rhizosphere and bulk soil at different depths. **(A)** The average relative abundance of the dominant bacteria phyla. **(B)** The average abundance of different Acidobacteria family in the bulk soil at 0–60 cm. **(C)** The average abundance of different Acidobacteria family in the rhizosphere at 0–30 cm. The significant difference in depth is indicated by small letters at P < 0.05.

Specific analysis was conducted to determine the differences in the composition of bacterial communities in the rhizosphere and bulk soil at different depths in a coastal saline-alkali citrus orchard. As shown in the results (**Figure 5A**), the average relative abundance of dominant bacteria in the rhizosphere was not significantly affected by depth, while the relative abundance of some dominant bacteria in the bulk was affected by depth. There was an increasing or decreasing trend of bacterial abundance in the vertical direction of the soil. Among them, the relative abundance of NC10 in the rhizosphere didn’t change with depth; however, in the bulk soil the average relative abundance of NC10 increased with increasing depth, i.e., from 1.33% to 12.69%, and the growth was particularly obvious in the 30–60 cm soil profile. In addition, the relative abundance of Acidobacteria, the dominant bacteria both in the rhizosphere and bulk soil second only to the Proteobacteria, in the bulk decreased significantly with increasing depth, i.e., from 20.69% to 11.58%. Its relative abundance in the rhizosphere had no significant difference at different depths, accounting for 11.75%–12.05%.

The relative abundances of the phylum Acidobacteria that consists of six families were analyzed in the rhizosphere and bulk soil. In the bulk soil, the average relative abundance of Ellin6075, mb2424, RB40, and Solibacteraceae decreased significantly with increasing depth, while the average relative abundance of Koribacteraceae and PAUC26f increased significantly with increasing depth (**Figure 5B**). Within a depth of 0–30 cm in the rhizosphere, the average relative abundances of the six families were not significantly different at different depths (**Figure 5C**).

We also analyzed the response of the six families in the Acidobacteria phylum to different environmental factors, and the results of the heat map showed the correlation. Ellin6075, mb2424, RB40, and Solibacteraceae were significantly positively correlated with nutrient-related variables, such as AvMn, SOM, AN, AvZn, and AK, and were negatively correlated with salinity and alkalinity -related variables, such as pH, ExCa, ExMg, Ca^2+^, Na^+^, and EC (**Supplementary Figure S5**). Based on the above results and combined with the physical and chemical properties of the rhizosphere and bulk soil, it could be concluded that the micro-environment formed by the citrus rhizosphere reduces the degree of salt-alkali stress for certain bacteria, and these bacteria can improve the nutrient availability in the citrus rhizosphere soil.

### 3.5 Responses of rhizosphere and bulk soil bacteria to environmental factors

**Figure 6:**
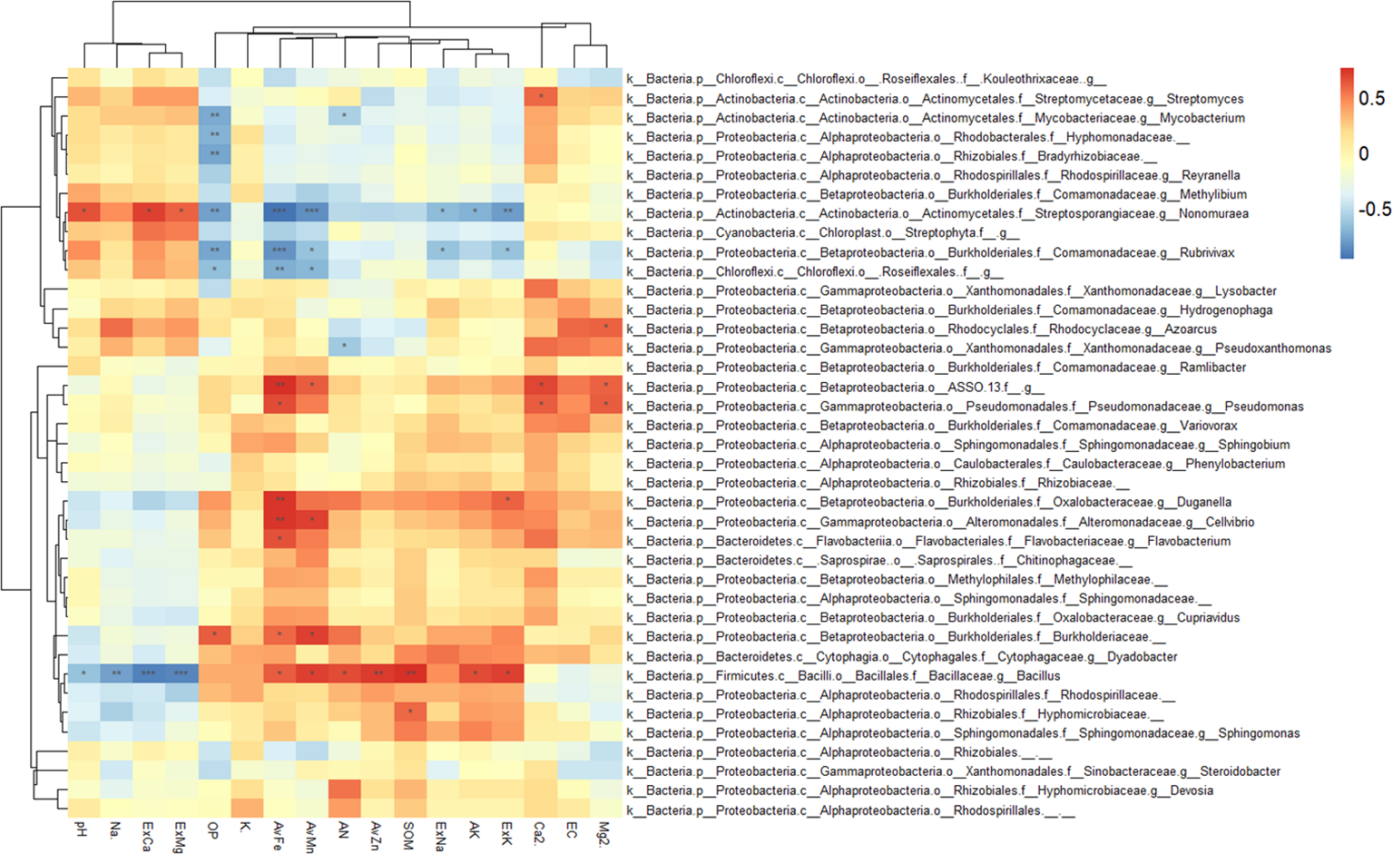
Linear discriminant analysis (LDA) of enriched bacterial species in rhizosphere and bulk soil.

The LDA based rhizosphere and bulk-enriched bacterial analyses are shown in **Figure 6**.

**Figure 7:**
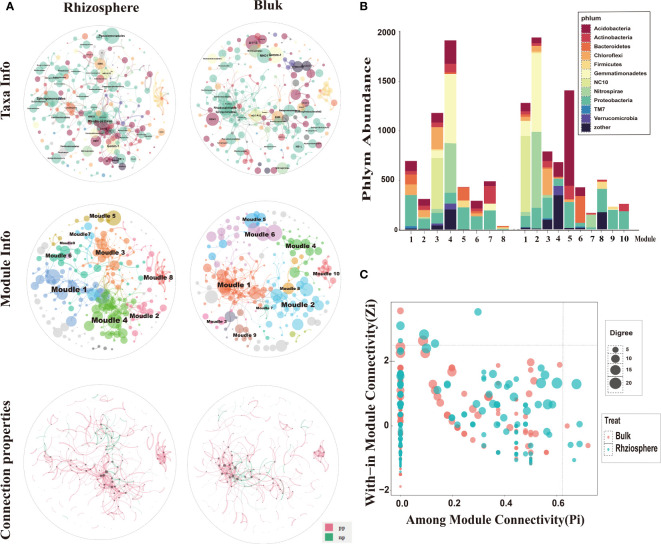
Heatmap showing the correlations between rhizosphere enriched bacteria and environmental factors. Asterisk indicates the correlation coefficient (*P < 0.05, **P < 0.01, ***P < 0.001).

The correlation analysis between enriched bacteria in the rhizosphere and environmental factors is shown in **Figure 7**. Most rhizosphere-enriched bacteria showed a positive correlation with nutrient variables, and some bacteria showed a significant or extremely significant positive correlation with nutrients. For example, Bacillus had a significant positive correlation with nutrients, such as SOM, AN, ExK, AK, AvZn, AvFe, and AvMn. However, it had a significant negative correlation with salinity, such as ExCa, ExMg, pH, and Na^+^. Burkholderiaceae had a significantly positive correlation with AvFe, AvMn, and OP. *Pseudomonas* had a significantly positive correlation with AvFe, Ca^2+^ and Mg^2+^, ASSO had a significantly positive correlation with AvFe, AvMn, Ca^2+^ and Mg^2+^, *Cellvibrio* had a significantly positive correlation with AvFe, AvMn, *Flavobacterium* had a significantly positive correlation with AvFe, and *Duganella* had a significantly positive correlation with AvFe and ExK. There are also a small number of enriched bacteria that were negatively correlated with nutrients and positively correlated with salinity. For example, *Nonomuraea* of the *Nephromyces* was significantly negatively correlated with available nutrients, such as AvFe, AvMn, OP, AK, ExK and ExNa, but significantly positively correlated with salinity, such as ExCa, ExMg, and pH. *Roseifflexales* were significantly negatively correlated with OP, AvFe and AvMn. *Rubrivivax* were significantly negatively correlated with OP, AvFe, AvMn, ExNa and ExK. Streptomyces were significantly positively correlated with Ca^2+^.

**Figure 8:**
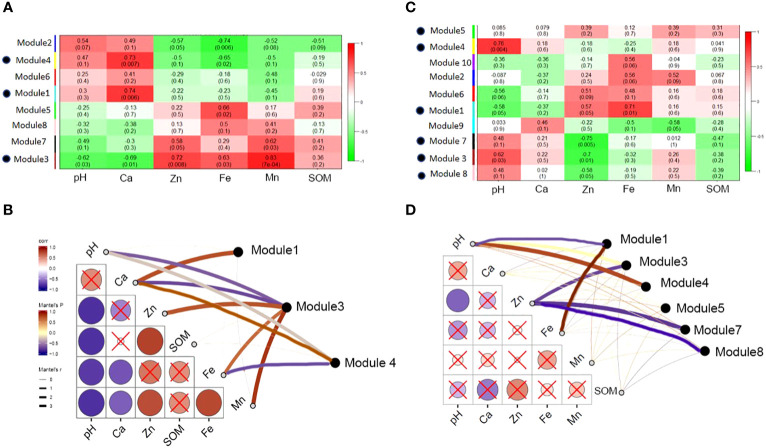
Heatmap showing the correlations between bulk soil enriched bacteria and environmental factors. Asterisk indicates the correlation coefficient (*P < 0.05, **P < 0.01, ***P < 0.001).

Consistent with the previous RDA results of the rhizosphere and bulk soil, most bulk soil enriched bacteria were negatively correlated with nutrients, but positively correlated with salinity (**Figure 8**). For example, GCA004 had a significantly positive correlation with ExCa, ExMg and Na^+^, while it had a significantly negative correlation with AvZn, K^+^, AK, ExK, SOM, AN. Wb1.A12 had a significantly positive correlation with ExCa, ExMg and Na^+^, while it had a significantly negative correlation with OP, AvZn, K^+^, AK, ExK, AN. BPC015 had a significantly positive correlation with ExMg and Na^+^, while it had a significantly negative correlation with AvZn, AK, ExK. PAUC26 had a significantly positive correlation with ExMg and Na^+^, while it had a significantly negative correlation with AK and ExK. Ellin6513 had a significantly positive correlation with ExCa and Na^+^, while it had a significantly negative correlation with AN. Nitrospiraceae had a significantly positive correlation with ExCa, ExMg and Na^+^, while it had a significantly negative correlation with AvZn, ExNa, K^+^, AK, ExK and AN.

In summary, most citrus rhizosphere-enriched bacteria were positively correlated with nutrient-related environmental variables, whereas bulk-enriched bacteria were positively correlated with saline-alkaline-related environmental variables. Enriched bacteria in the rhizosphere of citrus can promote nutrient availability and are tolerant to salt-alkali stress.

### 3.6 Differences in the ecological network of rhizosphere and bulk microorganisms and their responses to environmental factors

Previous results showed that the difference between the rhizosphere and bulk micro environment is the main factor affecting soil bacterial communities, and soil depth has a secondary effect on bacterial communities. Especially in the rhizosphere, the bacterial communities were not affected by depth. We constructed a random theory matrix (RMT) microbial ecological network of the citrus rhizosphere and bulk soil microbial community on the MENA website to analyze the differences in microbial interactions between the two soil compartments. The topological properties of the rhizosphere and bulk network structure are summarized in the **Supplementary Material** (**Supplementary Table S3**). The number of nodes in the rhizosphere (total nodes, 258) was greater than that of the bulk (231), the connection value between nodes in the rhizosphere (total links, 589) was greater than that in the bulk (477), the average degree of rhizosphere (avgK) and the average aggregation coefficient (avgCC) were greater than that for the bulk, and the average path length (GD) was less than that in the bulk (**Supplementary Table S3**). This shows that the connection between the network nodes in the rhizosphere was higher than that in the bulk. The information exchange of the bacterial communities in the rhizosphere was closer than that in the bulk soil. Bacterial interactions may form relatively stable rhizosphere communities.

**Figure 9:**
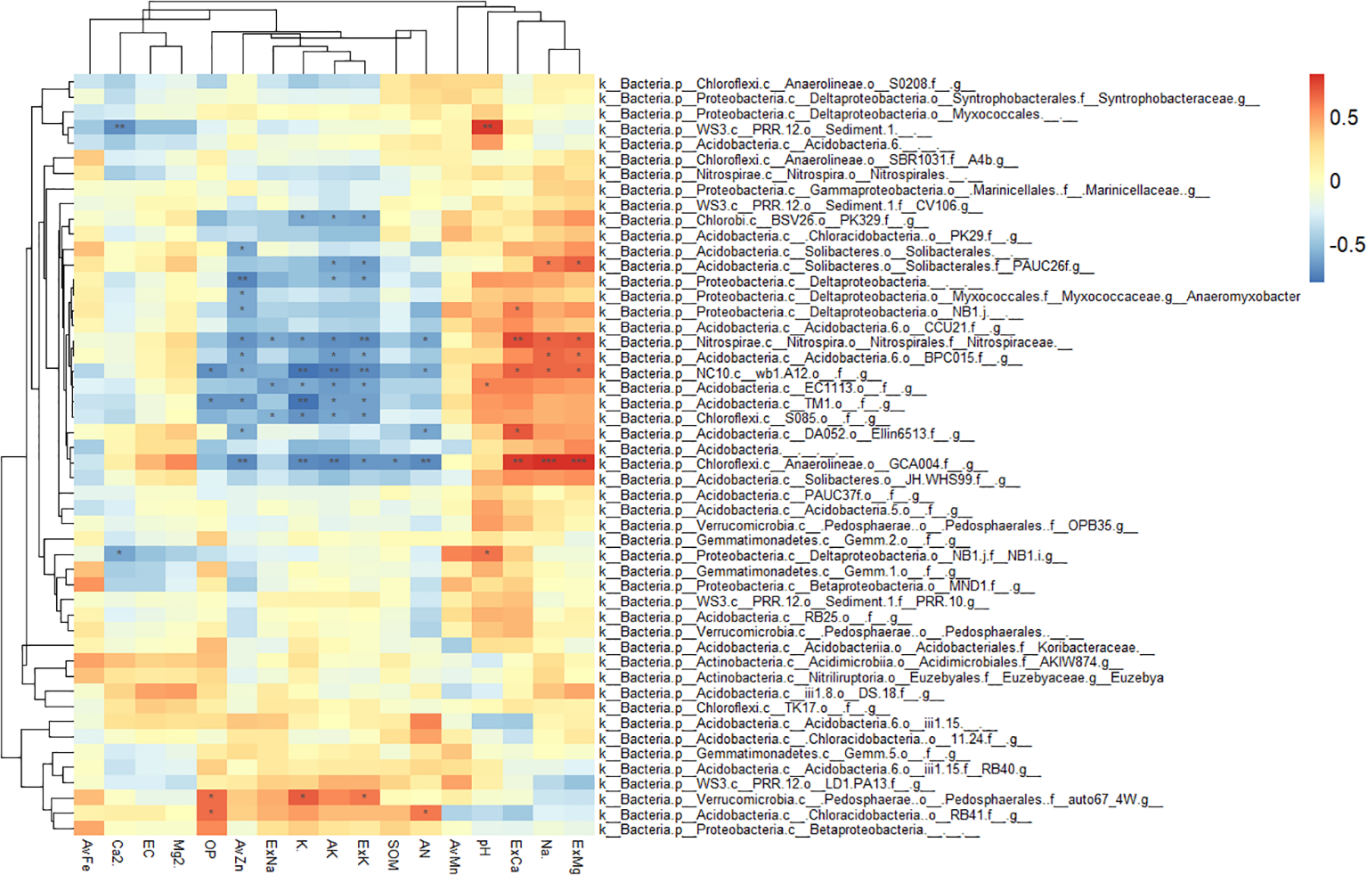
Microbial ecological networks and functional module structure characteristics of bacteria from rhizosphere and bulk soil. **(A)** The top two spheres indicate taxa information, and different colors correspond to different phyla. Each node represents an operational taxonomic unit (OTU), and node size represents abundance. The main enriched bacteria are labeled with names at the order level. The two middle spheres indicate module information, and different colors correspond to different main function modules. The two bottom spheres indicate the interaction between OTUs, where red indicates positive correlation and green indicates negative correlation. **(B)** Bacterial composition of the main functional modules of rhizosphere and bulk microbial ecological networks, expressed at the gate level. **(C)** ZP-plot showing the distribution of OTUs based on their module-based topological roles. Each dot represents an OTU in the bulk (red) or rhizosphere (green) dataset. The topological role of each OTU was determined according to the scatter plot of within-module connectivity (Zi) and among-module connectivity (Pi).

In the network composed of rhizosphere and bulk soil bacteria, Proteobacteria was the main phylum in the rhizosphere bacterial network, and in the bulk network both Proteobacteria and Acidobacteria phylum were dominant. Sphingomonadales, Rhodospirillales, and Pseudomonadales occupied a higher proportion in the rhizosphere and bulk networks. Clusters with more bacterial connections were divided into modules. The rhizosphere and bulk microbial community networks were composed of eight and ten main functional modules, respectively (node composition > 8). Rhizosphere and bulk nodes had different topological roles in the network. These nodes represent the roles of the key populations in or between the modules. The connectivity of most nodes in the rhizosphere and bulk soil was positive, and only a few nodes were negatively correlated. This indicates that in the overall network and functional modules of the rhizosphere and bulk, the connection between bacteria was mainly manifested as synergistic symbiosis (**Figure 9A**).

Specific analysis of the bacterial composition of the main modules (**Figure 9B**) showed that the bacterial compositions of the two soil compartments were quite different at the phylum level. In the rhizosphere, Proteobacteria was the main component of the sub-modules, followed by Actinomycota, Bacteroides, and *Chloroflexus*, which is consistent with the results of the rhizosphere bacterial community composition in this study. In addition, dominant bacterial phyla had the highest abundance in the module. For example, NC10 had the highest abundance in module 3, and Blastomonas and Nitrospira had the highest abundance in module 4. The main modules of bulk soil bacteria were Acidobacteria, Proteobacteria, and Bacteroides. Individual bacterial phyla were concentrated in some single sub-modules, such as NC10 bacterial phylum, which was the most abundant in module 1. The Bacillus phylum had the highest abundance in module 2 and the phylum Acidobacteria had the highest abundance in module 5. As explained above, the bacterial composition and functional modules of the rhizosphere and bulk soil bacterial networks were very different, and different dominant bacteria may lead to different module functions.

Most nodes in the network have connectivity within their respective modules, but the microbial populations corresponding to five nodes in the rhizosphere have strong connectivity between and within modules (Pi > 0.61, Zi > 2.2) (**Figure 9C**), indicating that certain bacteria in the rhizosphere not only participate in the internal connection of functional modules, but also connect modules with different functional modules, which are the key factors in regulating microbial networks. As explained above, in the rhizosphere and bulk microbial networks, communication between bacteria is mostly in symbiotic rather than competitive. The bacteria in the citrus rhizosphere are more closely connected, and there are key populations that can regulate the microbial network.

**Figure 10:**
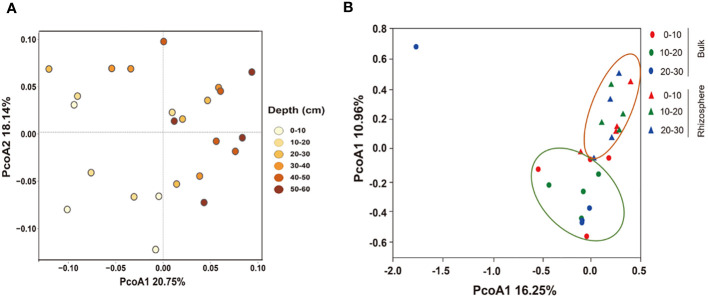
Correlation between main modules of rhizosphere and bulk network and soil environmental variables. **(A)** The correlation between the main functional modules of rhizosphere and soil environmental factors, the red color indicates highly positive correlation and green color indicates highly negative correlation; **(B)** the correlation between environmental factors and main rhizosphere modules with significant correlation; **(C)** the main functional modules of bulk correlated with soil environmental properties; and **(D)** the correlation between environmental factors and main bulk modules with significant correlation.

Based on the analysis of the environmental characteristics of the rhizosphere and bulk soil, differences in the response of the modules in the two networks to environmental factors were detected. The modules in the rhizosphere network are more closely related to environmental factors and are more strongly affected by soil environmental disturbances than those in the bulk soil. Module1, 2, 4 and 6 had positive correlation with pH and Ca, and negative correlation with Zn, Fe, Mn and SOM. Module 3, 5, 7 and 8 were the opposite. It is worth noting that modules 3 and 7 had similar functions. Module 3 was significantly positively correlated with the environmental variables related to soil available iron, zinc, manganese, and other nutrients and significantly negatively correlated with the environmental variables related to salt and alkalinity, such as pH and ExCa (**Figures 10A, B**). The response of module 3 to soil environmental variables was consistent with that of the rhizosphere-enriched bacteria to environmental variables. This shows that the bacterial community function of module 3 may be related to the absorption of nutrient elements, such as iron. Bulk network modules 3, 7 and 8 had positive correlation or significantly positive correlation with pH and ExCa, and negative or significantly negative correlation with AvZn, AvFe, and AvMn. Module1 had significantly positive correlation with AvFe and negative correlation with pH. Module 4 had significantly positive correlation with pH. (**Figures 10C, D**).

## 4 Discussion

### 4.1 Environmental changes with soil depth affected the composition of bulk soil bacterial communities

Previous research has indicated that there are significant differences in the structure of soil bacterial communities, as well as in pH, organic matter, and other nutrient variations, among different soil layers (Chao et al., 2012). This phenomenon could be attributed to the ability of bacteria to decompose organic matter and utilize nutrients such as carbon and nitrogen for growth and reproduction (Phillips et al., 2011; Str?M et al., 2005).In the vertical gradient of the soil profile, the physical and chemical properties of the rhizosphere and bulk soil were the same. The content of pH, EC, Na^+^, ExCa, and ExMg increased with depth, which is in line with the characteristics of saline soil (Jia et al., 2011). The content of effective nutrients, such as organic matter, nitrogen, phosphorus, potassium, zinc, iron, and manganese, decreases with an increase in soil depth, as they are affected by pH and salinity. There was obvious surface aggregation in the soil, which is consistent with the results of a previous study (Chunyan et al., 2007). In the vertical soil gradient, the pH increased significantly, and the bacterial communities decreased exponentially (Eilers et al., 2012). Alkaline-related variables, such as pH, were the main factors affecting bulk soil bacterial communities.

The 30–60 cm soil layer had high salinity, allowing salt-tolerant and halophilic bacteria (NC10) to accumulate. The relative abundance of NC10 increased with depth in the bulk soil (**Figure 5A**). NC10 is mainly composed of anaerobic halophilic bacteria that participate in anaerobic methane oxidation and nitrite denitrification. It has been detected in various hypoxic freshwater habitats and NO_2_-rich sediments in the South China Sea (Chen et al., 2015; Shen et al., 2015; Padilla et al., 2016). In shallow soil, bulk-enriched bacteria are closely related to saline-alkali environmental factors (**Figure 8**). These enriched bacteria, such as the WB1-A12 class of NC10 bacteria and Nitrospiraceae. were significantly positively correlated with salinity and pH, while they were significantly negatively correlated with nutrient elements. Studies have shown that Nitrospiraceae is enriched in the deep soil of rice growing regions, and their relative abundance significantly positively correlates with soil ExCa (Huang et al., 2014), which may play a role in salt tolerance in non-rhizosphere soil. In summary, changes in the soil environmental factors caused by depth influences the structure of the bacterial communities. Bacterial communities in deep soil (30–60 cm) are resistant to saline and alkaline conditions. The bacterial community in shallow soil (0–30 cm) prefers nutrients, and enriched bacteria, such as WB1-A12 and Nitrospiraceae, exist in the bulk soil to adapt to salt-alkali stress and have anaerobic and salt-tolerant properties.

### 4.2 Differences in bacterial community structure between rhizosphere and bulk soil

The availability of iron in coastal saline-alkali soils is affected by physical and chemical properties, such as pH, salinity, and organic matter, which are significantly different in the rhizosphere and bulk areas because of the effect of citrus roots (Dong et al., 2021). Especially in the vertical gradient of the soil profile, the change in soil properties is more complicated and is affected by many factors, such as plant absorption, biological cycle, soil parent material, and land usage (Jobbagy and Jackson, 2001).

Owing to the role of the citrus root system, the physical and chemical properties of the rhizosphere and bulk soil are different at the same soil depth. Owing to the application of organic fertilizer, root epidermal exudates, and root exudates (Phillips et al., 2009), citrus rhizospheres had higher C and N contents than those in bulk soil. C and N increase the metabolic rate, quantity, and related enzyme activities of soil bacteria, such as β-glucanase and *N*-acetyl-β-d-glucosidase (Drake et al., 2013). These enzymes decompose the organic matter to provide nutrients for microbial growth (Strom et al., 2005; Phillips et al., 2011) and release nitrogen and phosphorus, which can increase their effectiveness in the rhizosphere. In addition, citrus roots secrete a large amount of organic acid, such as citric acid, malic acid, phenolic compounds and flavonoids, to acidify the soil, which results in lower rhizosphere pH, ExCa, and ExMg content than those in the bulk soil (Dakora and Phillips, 2002; Cesco et al., 2010). These rhizosphere secretions also act as chelating agents to release metallic elements, such as zinc, iron, manganese, and copper, from immobilized compounds, thereby enhancing their solubility and mobility (Huang et al., 2005). In addition, bacteria secrete organic acids that participate in the activation of rhizosphere nutrients. Together, these factors cause differences in the physical and chemical properties of the rhizosphere and bulk soil (Bais et al., 2006).

The root system occupies the microbial habitat, and beneficial bacteria related to plant growth are screened out to reduce bacterial diversity in the rhizosphere (Shi et al., 2015). In addition, the root system affects the physical and chemical properties of the soil, forming a dynamic, stable, even, and nutrient-rich niche in the rhizosphere, which makes the rhizosphere bacterial community structure different from that of the bulk (**Figure 3B**) and weakens the differences caused by soil depths (DeAngelis et al., 2009).

By comparing the correlation between bacteria and soil environmental factors, it was found that the bacterial community in the rhizosphere was more related to the SOM, AN, OP, AK, AvZn, AvFe, AvMn, and other nutrient indicators, while the bulk soil bacterial community had a strong correlation with salt-alkaline environmental factors, such as pH, NA^+^, ExCa, and ExMg (**Figure 4B**). These results indicate that the interactions of bacteria and micro-environmental factors in the rhizosphere are important factors affecting the rhizosphere bacterial community (Xu et al., 2018b). In coastal saline soil, citrus roots secrete H^+^ and organic acids to decrease rhizosphere pH and chelate trace elements, such as zinc, iron, and manganese, to promote plant growth and development. Simultaneously, some root exudates become a source of bacterial nutrition (Bai et al., 2015) to enrich related bacteria (Bais et al., 2006; Haichar et al., 2008; Dennis et al., 2010). Subsequently, enriched bacteria can participate in the activation of soil nutrient elements, which are further absorbed and utilized by plants and promote the reproduction of bacterial communities (Chen et al., 2014; Meyer et al., 2018; Deng et al., 2019; Deng et al., 2020). In conclusion, because of the effect of plant root exudates, the bacterial community in the citrus rhizosphere was greatly affected by nutrient elements, whereas that in the bulk soil was mainly affected by salinity-related variables. The correlation between the bacteria and soil nutrients was as follows: rhizosphere region > bulk 0–30 cm > bulk 30–60 cm.

The dominant bacterial phyla in the rhizosphere and bulk soil were Proteobacteria, Acidobacteria, Gemmatimonadetes, and Actinobacteria. These bacterial phyla are also dominant in other soil environments (Bulgarelli et al., 2015; Xu et al., 2018a). In this study, the average relative abundance of Acidobacteria in the bulk soil was higher than that in the rhizosphere, which is consistent with the results of previous studies (Trivedi et al., 2012), and its relative abundance decreased significantly with the increase in soil depth. There were no significant differences with depths observed in the rhizosphere. Acidobacteria is subdivided into six families, among which the relative abundance of Ellin6075, MB2424, RB40, and Solibacteraceae in bulk decreased significantly with increasing depth, but there was no significant difference observed in the rhizosphere (**Figure 5**), which indicated that the special microdomain environment formed in the rhizosphere region not only affected the community structure of bacteria, but also protected them from salt stress. Many studies have shown that plants closely interact with microorganisms through the release of signals from their roots (Rajkumar et al., 2010; Singh et al., 2010; Meena et al., 2017). The presence of roots weakens the influence of soil depth on the structure and composition of the bacterial communities (Eilers et al., 2012; Kumar et al., 2016). The four mentioned bacteria were significantly positively correlated with nutrient variables, such as AvMn, SOM, AN, AvZn, and AK (**Supplementary Figure S5**). As an organic heterotrophic organism, Ellin6075 has been shown to have a significant positive correlation with tryptophan (Zhang et al., 2019). Solibacteraceae has been shown to be involved in the resistance of some fungal pathogens (*Fusarium oxysporum*) (Mendes et al., 2013), and they are also reported to participate in the carbon cycle of the soil (Zhang et al., 2019). It can be shown that the environment formed by the citrus root alleviates the inhibition of these bacteria by saline-alkali conditions, and these bacteria have a promoting effect on the nutrient availability of the citrus rhizosphere soil compared to that of the bulk soil.

### 4.3 Response of rhizosphere and bulk soil enriched bacteria to soil environment

Minimal discriminant analysis (LDA) was used to screen out rhizosphere and bulk soil enriched bacteria (**Figure 6**) and to analyze their correlation with soil environmental factors. From the results of the correlation heat map, the enriched bacteria in the rhizosphere had a strong correlation with the nutrient variables (**Figure 7**), whereas the enriched bacteria in the bulk soil strongly correlated with the saline-alkali variables (**Figure 8**). The presence of enriched bacteria in the rhizosphere of citrus plants can promote the availability of iron and other nutrients. For example, *Pseudomonas* can produce various siderophores, which can increase the availability of iron in the soil (Cornelis, 2010). *Pseudomonas* was also a promising bioinoculant having plant growth-promoting traits, which promotes growth and development in A. paniculata and may be applied to other plants also (Thakur and Yadav, 2023a). Cellvibrio Bin79 can store iron at a concentration higher than the soluble limit of Fe^+^ in bacteria (Rivera, 2017). Certain strains of *Duganella* produce different siderophores (Akob et al., 2014). Streptomyces was thermotolerant and halotolerant (Thakur and Yadav, 2023b). Bacillus, which can reduce Fe (III) to Fe (II) under *in vitro* conditions (Ghorbanzadeh et al., 2014). Bacillus Species from Maize Rhizosphere also carried genetic elements include those of siderophore production and iron acquisition (Olanrewaju et al., 2021).

### 4.4 Differentiation of rhizosphere bacterial network under the influence of soil environment

From the network analysis results, it can be seen that the rhizosphere microbial network had more nodes and associations than that in the bulk soil, and the interaction of bacteria in the rhizosphere network was closer and more complex (**Supplementary Table S3**), which is consistent with previous studies (Shi et al., 2016; Yan et al., 2017; Zhang et al., 2018). In this study, the network of citrus rhizosphere bacteria had stronger connections within and between modules than those in the bulk. The interaction of the rhizosphere modular community was closer than that of the bulk community. There are “all-rounder” colonies within and between the modules in the rhizosphere (**Figure 9**). These colonies play a key regulatory role in the rhizosphere microbial network connection (Deng et al., 2011). This indicates that when the microbial information exchange in the rhizosphere network is closer, the energy transfer is more efficient, the interaction between the bacteria is stronger, and the shared niche is bigger (Faust and Raes, 2012; Berry and Widder, 2014; Zelezniak et al., 2015; Deng et al., 2016). In addition, the average path length (GD) of the citrus rhizosphere microbial network was smaller than that of the bulk network. Studies have shown that networks with small path lengths are considered “small world” networks (Watts and Strogatz, 1998; Boccaletti et al., 2006). By analyzing the correlation between the soil physical and chemical properties and bacterial communities in this study, we believe that the “small world” network of the rhizosphere may be related to the response of soil nutrient disturbances (Zhou et al., 2010; Banerjee et al., 2018). In the rhizosphere, various nutrients, such as iron, accumulate in the roots due to the absorption of nutrients by citrus roots. The disturbance of nutrients may lead to a closer symbiotic and competitive relationship between the rhizosphere bacteria. Therefore, the nutrient requirements of citrus roots make information exchanges between the bacteria in the rhizosphere network more frequently than those by bulk soil bacteria, and these exchanges are hardly affected by environmental factors, such as salinity and alkalinity (Deng et al., 2011; Zhou et al., 2011; Ma et al., 2016).

Highly and closely connected bacteria in the network are called modules, which are individual clusters with specific functions (Luo et al., 2006; Bissett et al., 2013). Studies have shown that modules can be used as indicators of microbial niches and the bacteria in these modules share similar niches (Eilers et al., 2012; Wu et al., 2016; Zhang et al., 2018). There are more modules in the bulk soil than in the rhizosphere, which indicates that the microbial niche in the bulk is greater than that in the rhizosphere. The bulk soil is considered to be an incoherent habitat for bacteria; in contrast, the rhizosphere has a more even soil environment owing to the existence of the root system, thereby reducing the niche of bacteria (Fierer and Lennon, 2011; Fan et al., 2017).

The rhizosphere and bulk network modules responded differently to the soil environmental variables. Module 3 in the rhizosphere had the strongest response to soil environmental variables, which was positively correlated with the available nutrients, such as iron, zinc, and manganese, and negatively correlated with calcium and pH. Module 7 was similar to Module 3. Rhizosphere modules 1 and 4 and bulk modules 3, 4, 5, 7, and 8 showed the opposite results (**Figure 10**). The correlation between the module and environment also proves the role of habitat heterogeneity in the formation of the module (Olesen et al., 2007). Rhizosphere-enriched bacteria were tolerant to salinity and beneficial to the plants in modules 3 and 7 (**Supplementary Table S4**). For example, Streptomyces can produce siderophores to improve the absorption of certain nutrients by plants, and the secreted indole acetic acid can further promote plant growth (Huang et al., 2014). Some strains of Mycoplana have nitrogen-fixation functionality. *Thalassobaculum* and *Woodsholea* both exhibit salt tolerance (Zhang et al., 2008; Urios et al., 2010), and *Woodsholea* is also related to the hydrolysis of organic matter. These rhizosphere-enriched bacteria, which are tolerant to salt and beneficial for plant iron and other nutrient absorption play an important role in the rhizosphere bacterial community.

## 5 Conclusion

This study is based on the phenomenon that the “Beni-Madonna” citrus growing in the coastal saline-alkali land often lacks iron and other micronutrients. We used 16S rRNA gene amplicon technology to explore the response of the citrus soil bacterial communities to environmental factors in the rhizosphere and bulk soil. The main conclusions are as follows: (1) Compared to the bulk soil, the citrus rhizosphere microenvironment reduces the degree of salt-alkali stress of certain bacteria, and these bacteria maybe promote the activation of citrus rhizosphere soil nutrients. (2) Citrus rhizosphere and bulk soil bacterial communities are differently affected by soil environmental factors. The rhizosphere bacterial communities are mainly affected by nutrient-related environmental factors, while the bulk soil bacterial communities are mainly affected by saline alkali-related environmental factors. Bulk soil communities are increasingly affected by soil salinity and alkalinity as the soil depth increases. The correlation between bacterial communities and nutrient environmental factors were as follows: rhizosphere area > bulk 0–30 cm soil profile > bulk 30–60 cm soil profile. The relationship between bacterial communities and saline-alkali related environmental factors shows the opposite trend. (3) In the coastal saline-alkali citrus orchard soil, an increase in depth led to an increase in the content of saline-alkaline-related elements in the soil. Changes in the soil environmental factors caused by depth influences the structure of bacterial communities. The bacterial communities in the deep soil profiles are resistant to saline and alkali, while the bacterial communities in the shallow soil profile prefer nutrients. Enriched bacteria, such as WB1-A12 and Nitrospiraceae, exist in the non-rhizosphere area maybe have the ability to adapt to salt-alkali stress and the function of anaerobic salt tolerance. (4) Citrus roots were distributed in a shallow soil profile (0–30 cm) in the coastal saline-alkali land. The citrus roots are rich in some salt-alkali stress bacteria (such as *Thalassobaculum* and *Woodsholea*) and fix nitrogen (*Mycoplana*). We hypothesize that *Pseudomonas*, *Cellvibrio*, *Duganella*, and *Streptomyces* secrete siderophores and plant hormones to promote the absorption of iron and other nutrients and enhance their ability to alleviate stress by citrus.

## Data availability statement

The original contributions presented in the study are included in the article/**Supplementary Files**, further inquiries can be directed to the corresponding author/s.

## Author contributions

TJ: Conceptualization, Data curation, Formal analysis, Investigation, Methodology, Software, Validation, Visualization, Writing – original draft, Writing – review & editing. JC: Data curation, Formal analysis, Software, Writing – review & editing. YH: Data curation, Resources, Writing – review & editing. XC: Conceptualization, Data curation, Formal analysis, Investigation, Writing – review & editing. YW: Resources, Writing – review & editing. GL: Resources, Writing – review & editing. RW: Software, Visualization, Writing – review & editing. KX: Data curation, Writing – review & editing. LL: Supervision, Writing – review & editing. HL: Funding acquisition, Resources, Writing – review & editing. ST: Project administration, Resources, Supervision, Writing – review & editing.
