# Supplementary material for: Characteristics of bacterial communities in rhizosphere and bulk soil in Fe-deficient citrus growing in coastal saline-alkali land
Source: Front Plant Sci. 2024 Feb 20;14:1335843. doi: 10.3389/fpls.2023.1335843 (PMC10914252; doi:10.3389/fpls.2023.1335843)
Supplement: Supplementary file 1 [file DataSheet_1.docx]

**Supplementary Material**

Characteristics of bacterial communities in rhizosphere and bulk soil in Fe-deficient citrus growing in coastal saline-alkali land

Tianchi Jiang ^a^†, Jiuzhou Chen ^a^†, Yu Huang ^b^†, Xiaoyan Chang ^a^†, Yuping Wu ^c^, Gaoping Liu ^d^, Runze Wang ^a^, Kuan Xu ^a^, Lingli Lu ^a^, Haizhong Lin^e^* and Shengke Tian ^a^*

**Author affiliations and addresses:s**

^a^MOE Key Laboratory of Environmental Remediation and Ecological Health, College of Environmental and Resource Sciences, Zhejiang University, Hangzhou 310058, China

^b^Xiangshan Agricultural and Rural Bureau, Ningbo, China

^c^Ningbo Agricultural and Rural Bureau, Ningbo, China

^d^Huangyan Agricultural and Rural Bureau, Taizhou, China

^e^Agricultural Technology Extension Center of Huangyan District, Taizhou, China †These authors contributed equally to this work.

***Corresponding author:**

MOE Key Laboratory of Environmental Remediation and Ecological Health, College of Environmental and Resource Sciences, Zhejiang University, Hangzhou 310058, China. E-mail: [tiansk@zju.edu.cn](mailto:tiansk@zju.edu.cn). Tel.: +86-571-88982515, Fax: +86-157-88982907.

Agricultural Technology Extension Center of Huangyan District, Taizhou 318020, China. E-mail: [hyhaizi@163.com](mailto:hyhaizi@163.com). Tel.: +86-0576-84219351.

**Summary of the numbers in supporting information:**

Number of pages: 4

Number of Tables: 3

**Table S1.** Physicochemical properties of the soil collected from the town of ShiPu in Ningbo City, Zhejiang Province, China

| **Analysis index** | **Value** |
| --- | --- |
| pH | 7.64±0.14 |
| EC (ds·m^-1^) | 1.70±0.09 |
| TC (%) | 1.43±0.01 |
| TN (g·kg^-1^) | 1.29±0.17 |
| K (g·kg^-1^) | 13.55±2.05 |
| Ca (g·kg^-1^) | 12.82±1.38 |
| Mg (g·kg^-1^) | 15.16±1.43 |
| Na (g·kg^-1^) | 9.62±0.43 |
| Zn (mg·kg^-1^) | 138.95±15.83 |
| Fe (g·kg^-1^) | 25.70±1.15 |
| Mn (mg·kg^-1^) | 930.27±95.92 |
| Cu (mg·kg^-1^) | 32.69±1.90 |

**Table. S2**. Salt anion content in the rhizosphere and bulk of coastal soil profiles. Significant differences in soil depth are indicated by small letters (P < 0.05); significant difference between rhizosphere and bulk soil is indicated by asterisks (* < 0.05, ** < 0.01)


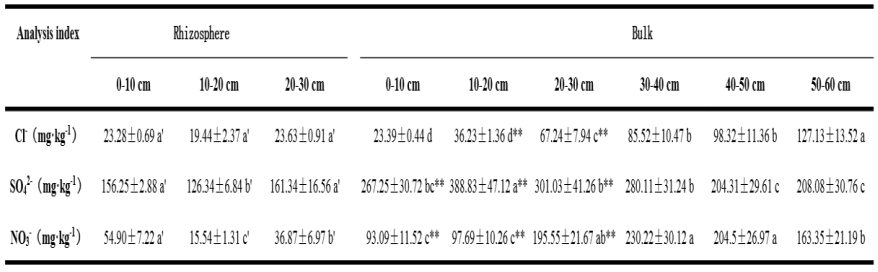


**Table S3.** Network topological indices of bacterial communities in the rhizosphere and bulk soil


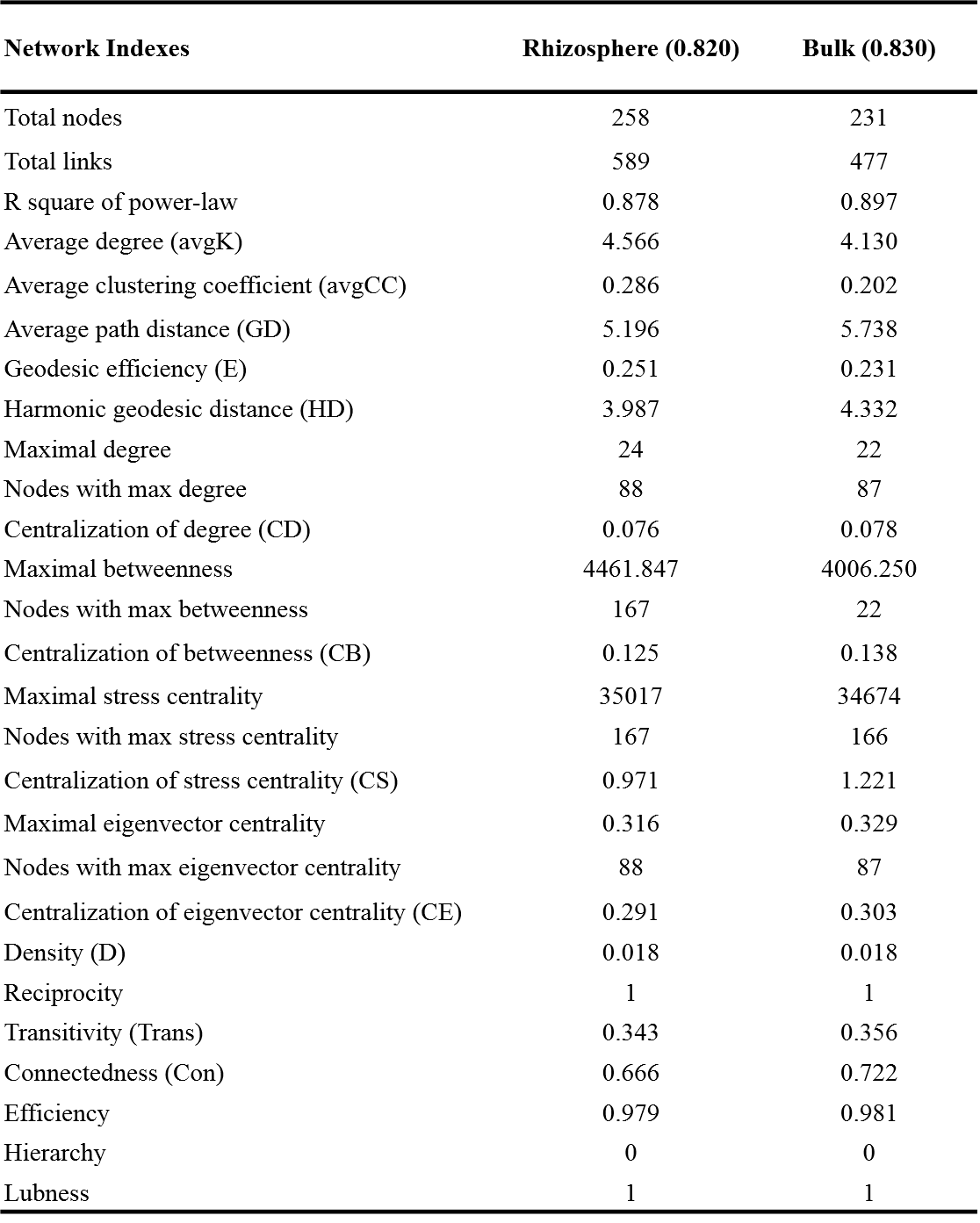


**Table S4**. Bacterial composition of functional modules in the rhizosphere in response to soil nutrient variables

| **Module** | **Phylum** | **Class** | **Order** | **Family** | **Genus** |
| --- | --- | --- | --- | --- | --- |
| **Module 3** | Proteobacteria | Alphaproteobacteria | Rhodospirillales | Rhodospirillaceae | Skermanella |
|  | Proteobacteria | Alphaproteobacteria | Rhodospirillales | Rhodospirillaceae | Thalassobaculum |
|  | Proteobacteria | Alphaproteobacteria | Rhodobacterales | Hyphomonadaceae | Woodsholea |
|  | Proteobacteria | Alphaproteobacteria | Caulobacterales | Caulobacteraceae | Mycoplana |
|  | Proteobacteria | Betaproteobacteria | Burkholderiales | Comamonadaceae | Hydrogenophaga |
|  | Proteobacteria | Betaproteobacteria | Burkholderiales | Comamonadaceae | Methylibium |
|  | Proteobacteria | Betaproteobacteria | Burkholderiales | Burkholderiaceae | NA |
|  | Actinobacteria | Actinobacteria | Actinomycetales | Streptomycetaceae | Streptomyces |
|  | Chloroflexi | Chloroflexi | Roseiflexales | NA | NA |
|  | Firmicutes | Bacilli | Bacillales | NA | NA |
| **Module 7** | Proteobacteria | Alphaproteobacteria | Rhodospirillales | NA | NA |


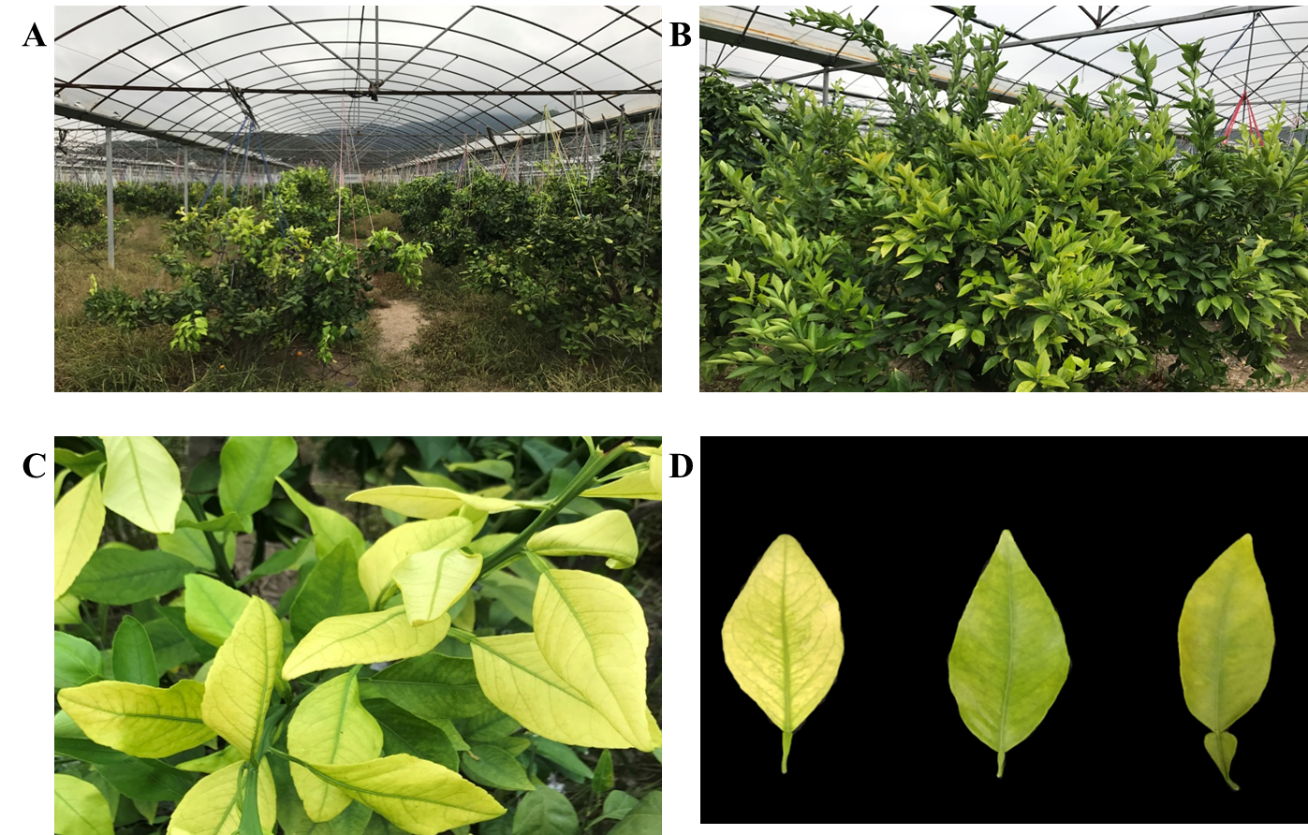


**Figure S4.** Citrus trees in saline-alkali land showed obvious iron deficiency yellowing.


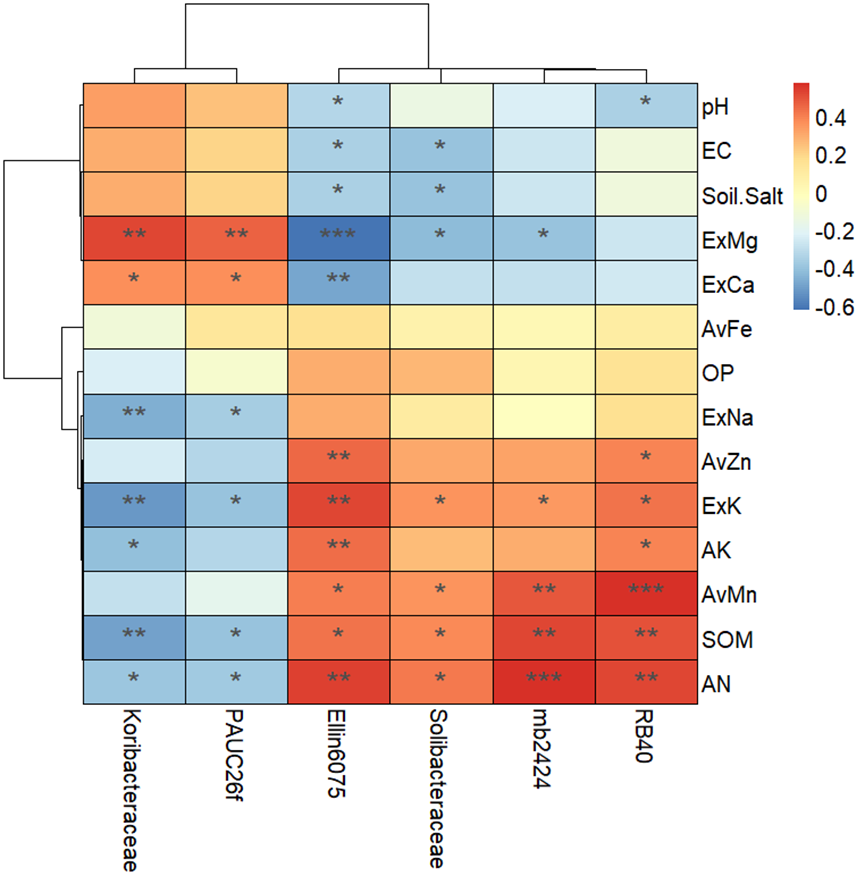


**Figure S5.** The heatmap shows the correlations between different Acidobacteria family and environmental factors. Asterisk indicates the correlation coefficient (*P < 0.05, **P < 0.01, ***P < 0.001).
